# Influence of Alloying Elements on the Mechanical Properties of Anodized Aluminum and on the Adhesion of Copper Metallization

**DOI:** 10.3390/ma14227028

**Published:** 2021-11-19

**Authors:** Oleg S. Medvedev, Ekaterina E. Alyasova, Rona E. Besprozvannaya, Asadula A. Gadzhiev, Veronika V. Krivova, Andrey S. Kondratev, Artem E. Kim, Pavel A. Novikov, Anatoliy A. Popovich

**Affiliations:** 1 LLC “Rusoxide”, 143026 Moscow, Russia; aee@rusoxide.ru (E.E.A.); bre@rusoxide.ru (R.E.B.); gaa@rusoxide.ru (A.A.G.); kvv@rusoxide.ru (V.V.K.); kas@rusoxide.ru (A.S.K.); 2 Peter the Great Saint-Petersburg Polytechnic University, 195251 Saint Petersburg, Russia; artem_7.kim@mail.ru (A.E.K.); novikov_pa@spbstu.ru (P.A.N.); director@immet.spbstu.ru (A.A.P.)

**Keywords:** anodic aluminum oxide, adhesion, insulated metal substrate

## Abstract

The active development of the power electronics market and a constant increase in the prices of components require new materials and approaches, including a power module packaging technology. The use of aluminum instead of copper in the power module baseplate is an interesting and promising solution. The insulated metal baseplate is one of the most extensively developed technologies nowadays. The object of this study is an insulated metal substrate based on anodized aluminum. The main goal of the article is the comparison of copper topology adhesion to an anodized aluminum oxide layer formed on different aluminum alloys with aluminum content of at least 99.3 wt %. Peel test and pull-off adhesions showed a twofold difference for both aluminum alloys. The high ordered defect-free anodized alumina formed on alloys with copper content of 0.06 wt % had a mean pull-off adhesion of 27 N/mm^2^ and hardness of 489 HV. In the case of the alloy with copper content of around 0.15 wt %, it had hardness of 295 HV and a mean pull-off adhesion of 12 N/mm^2^. The results of our microstructure investigation showed that anodized alumina based on alloys with copper content of around 0.15 wt % is fragile due to spherical holes. Summing up the results, it can be concluded that not all initial impurities are critical for anodized alumina, but some, specifically copper, dramatically decreased the mechanical properties of anodized alumina.

## 1. Introduction

The continuous trend of increasing the efficiency of electricity conversion technology, as well as the rapid growth of the development of the electric vehicle market, actively stimulates the development of the power electronics market. The main trend in the development of power electronics is the replacement of the traditional metal oxide semiconductor field effect transistors with an insulated gate (MOSFET) with high-power insulated gate bipolar transistors (IGBT) in many applications at medium voltage and uncontested applications of IGBTs for high voltage levels. The largest growth is shown by the segments of industrial drive, high-power direct current (HVDC), and electric vehicles. Currently, market leaders of power electronics (Infineon, ABB, Mitsubishi, etc.) are changing both the design and the materials used in the assembly of power modules to meet the needs of the growing electronics market [1,2]. At the moment, the most innovative products on the market are IGBT power modules from Mitsubishi, which combine the newest silicon semiconductor chips and a packaging approach with new materials and composites [2,3,4,5]. The comprehensive review of the current status and future trends of power modules is presented in [6]. The realities of the modern market require, on the one hand, an increase in the quality and reliability of power modules, and on the other hand, a reduction in the cost of the final product. One of the acute issues of the materials used is in the packaging of power modules due to the constant increase in the prices of baseplate material, such as copper. It is clear that aluminum is cheaper, but it has own specific features. From a reliability standpoint, improvements in insulation reliability and structural reliability in general are required for the power module baseplate. For example, the idea of using direct bounded aluminum (DBA) ceramics instead of traditional direct bounded copper (DBC) ceramics has long been considered by manufacturers and research laboratories. Thus, it was shown in [7] that the thermal impedance of DBA substrates is inferior to only 10–15% of DBC substrates, while DBA substrates are more stable with active and passive thermal cycling, which does not lead to the delamination of metal plates.

The alternative to using metallized ceramics for power electronic applications is to use insulated metal substrate (IMS). The use of pure polymers as an insulating layer is not justified due to low thermal conductivity. The dielectric polymer/ceramic mixture is currently the most common solution. Despite the relatively low thermal conductivity of 2–10 W/mK [8,9,10] to compare with traditional ceramics, the possibility of using thin layers of a polymer composite allows achieving thermal impedance values of the composite substrate that meet the requirements of power module manufacturers. The enhanced transient thermal performance [9,11,12] and the increased thermal cycling capability of IMS [4,5] can be seen as major advantages over DBC solutions for high-power modules. Copper is the most widely used metal as the baseplate for IMS solutions in power electronics. However, the idea of replacing the copper baseplate of a power module in order to reduce the cost and weight remains a challenge task for manufacturers of power modules. Aluminum as a heat-dissipating substrate has found wide applications in LED technology. One of the ways to use aluminum as a baseplate is the creation of a composite substrate, where anodic aluminum oxide is grown on an aluminum baseplate to provide the necessary dielectric strength. A printed circuit board (PCB) is formed by means of a galvanic copper process with subsequent etching of the necessary topology. In addition to the above advantages, this solution eliminates the step of DBC soldering on the baseplate.

Anodizing is a very common method of producing aluminum oxide film, which is characterized by low energy consumption and a wide range of functional properties. From a functional point of view, the anodic film has wear resistance and electrical insulating properties. In Ref. [13], an anodic aluminum oxide (AAO) layer is used as a barrier layer to prevent the migration of copper ions, an adhesive sublayer for epoxy resin, and finally as an additional electrical insulating layer. For the widespread introduction of anodized aluminum substrates in power modules, it is necessary to develop technology based on readily available branded aluminum alloys. However, the properties of the anodized layer vary considerably from alloy to alloy [14,15,16,17]. One of the significant parameters on which we focused in this work is the adhesion of copper metallization to AAO and the mechanical strength of the oxide. Compliance with the stringent requirements of manufacturers of power modules for this parameter is justified by high requirements for the resource of power modules. Moreover, in the past decade, this parameter has received increased attention, caused by the development of the electric vehicle market and the increased vibration load on the power modules. This paper compares the adhesive/cohesive strength of metallization on AAO obtained on two readily available “commercially pure” aluminum alloys of A1100 series. The paper presents the results of a study of peel test adhesion and pull-off adhesion, two important parameters controlled by power module manufacturers. The results of adhesion studies are correlated with the micro hardness of AAO and its structure, which was studied by scanning electron microscopy.

## 2. Materials and Methods

In this work, two aluminum alloys (AA) of A1100 series with the names AA#1 and AA#2 were investigated. The results of the elemental composition measured by atomic emission spectroscopy (ARGON-5SF, Spectrosoft, Troitsk, Russia) are given in Table 1. The sensitivity of this method for all presented chemical elements was better than 0.002 wt %. The total content of Mg, Mn, Zn, Cr, Ti and Ni impurities in these alloys was less than 0.05 wt %. Furthermore, the names of the investigated AAO correspond to the used initial alloys.

Aluminum blanks with a size of 320 mm × 240 mm × 3 mm were mechanically cleaned and then degreased before anodizing. Direct anodizing was carried out in an oxalic acid 3 wt % with a voltage stabilization at 120 V at temperature 15 °C until an anodized layer thickness of about 70–80 µm was reached. After anodizing, the blanks were moved according to the manufacturing process of substrates for power modules developed by LLC Rusoxide. The blanks were filled with dielectric lacquer followed by polymerization. After polymerization, mechanical cleaning of the surface from the remaining lacquer was applied, as well as for the planarization of the surface. Magnetron sputtering of targets with a purity of no less than 99.99% was used to deposit an adhesive chromium sublayer and sputtering copper layer. The deposition chamber was preliminarily evacuated to a vacuum of 2 ×10^−3^ Pa. The deposition process itself proceeded with the supply of argon, and a pressure in the chamber of 0.4 Pa. Then, 100 or 300 µm of copper was galvanically deposited on vacuum metallization, and copper was etched according to the test coupon topology. To increase adhesion, additional annealing was carried out at a temperature of 300 °C for 120 min.

**Figure 1:**
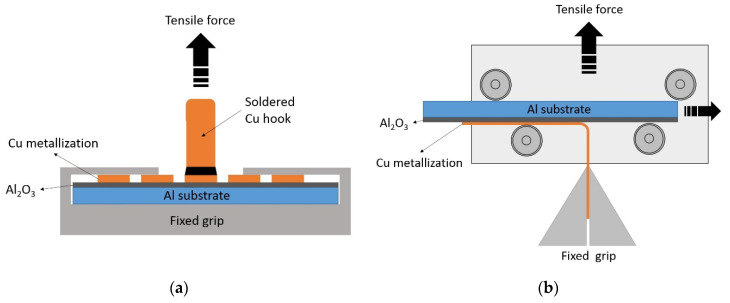
(**a**) Scheme of pull-off adhesion measurements. (**b**) Scheme of peel test adhesion measurement.

The adhesion strength between copper metallization and AAO surface was evaluated by performing pull-off and peel tests. Tests were executed in accordance with the IEC 62326-4-2013 standard using a slow tensile testing machine (RKM 0,5.1, Etalon-Profit, Ivanovo, Russia). Figure 1a shows a scheme of an experiment to measure a peel-off adhesion. A copper hook was soldered to a contact pad with a diameter of 3 mm. A pull-off test was carried out 20–30 min after soldering. A sample with a soldered hook was fixed in a rigidly fixed grip, and a copper hook engaged with the force gauge. The displacement speed of the force gauge in pull-off experiments was 35 mm/min. Figure 1b shows a schematic of an experiment to measure a peel test adhesion. In this experiment, the sample was placed in a movable mount attached to the force gauge. The sample in this tooling can freely slide to the sides along the rollers. A free edge of copper track is clamped into a fixed grip. The traverse speed in this experiment was also 35 mm/min.

The cross-section and morphology were studied using a field emission scanning electron microscope (Tescan Mira3, Brno, Czech Republic) with an operating voltage of 10 kV in the backscattered electron detection mode.

## 3. Results and Discussion

**Figure 2:**
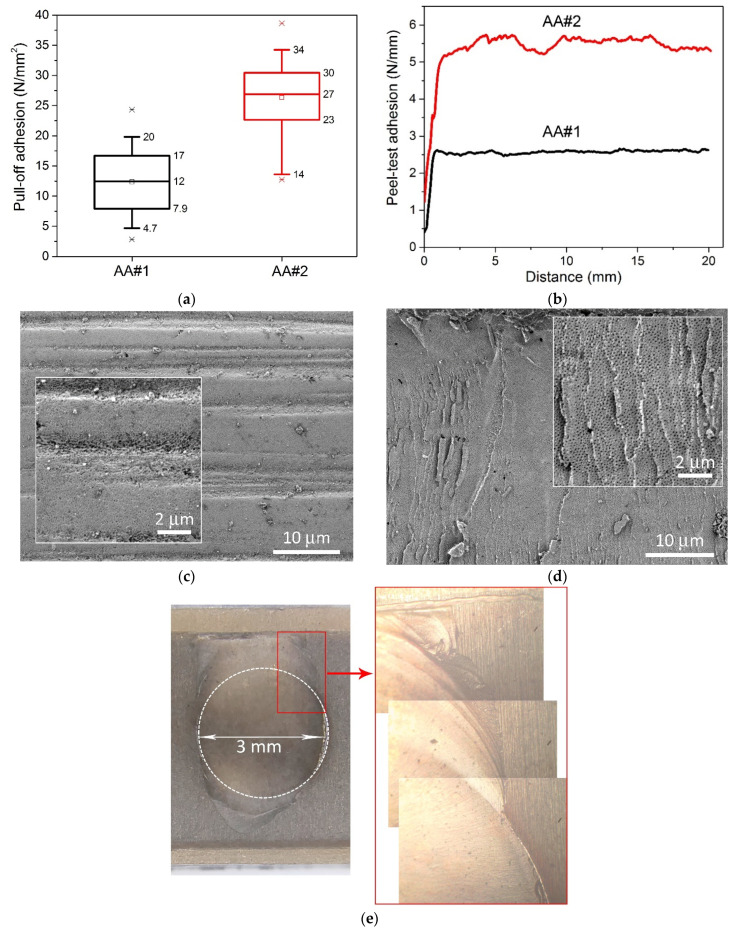
(**a**) Results of pull-off adhesion of 300 µm copper for AA#1 and AA#2 aluminum alloys. (**b**) Peel test adhesion results of 100 µm copper for AA#1 and AA#2 aluminum alloys. (**c**) SEM images of AAO surface planarized in the grinding machine. (**d**) SEM images of AAO surface after adhesion tests. (**e**) Optical images of cohesion failure after pull-off test of AA#2.

Figure 2 shows the results of measurements of pull-off adhesion of (a) 300 μm copper and (b) peel test of 100 μm copper. Pull-off adhesion was measured at more than 50 points evenly spaced over an area of 330 × 240 mm^2^. For AA#1, the average and median values were 12 N/mm^2^, while for AA#2, they were 27 N/mm^2^. SEM images of planarized AAO surface are shown in Figure 2c. The morphology was the same for both type of samples. The horizontal grooves were created by flattening the initial surface of the AAO with the abrasive brushes of the grinding machine. It should be noted that almost all the pores of the oxide closed after the planarization. Figure 2d demonstrates the typical morphology of cohesive failure of AAO after adhesive tests, identical for both sample types. The layer of closed pores was torn off along with metallization, and open pores are observed as a result of a cohesive destruction. We observed 30–80% cohesive failure within the AAO volume after pull-off of 3 mm copper contacts for the AA#1 sample, and 80–100% cohesive failure for the AA#2 sample. A distinctive fact of sample AA#2 is the propagation of cohesive failure beyond the boundaries of metallization (see Figure 2e), which indicates the mechanical integrity and strength of the entire AAO layer.

Figure 2b shows the results of peel test adhesion measurements of 100 μm thick copper. In the case of AA#1, the adhesion value was 2.5 N/mm, and for AA#2, 5.5 N/mm. In both cases, cohesive failures of the copper metallization from the AAO were also observed throughout the peeling process. Both types of adhesion experiments showed around two times the difference of cohesive strength of AAO on different aluminum alloys from the A1100 series. A similar trend was observed for various anodizing modes, indicating the absence of an optimal mode for any of the alloys, and the adhesive/cohesive properties are determined exclusively by the initial amount of certain dopant in the aluminum alloy.

**Figure 3:**
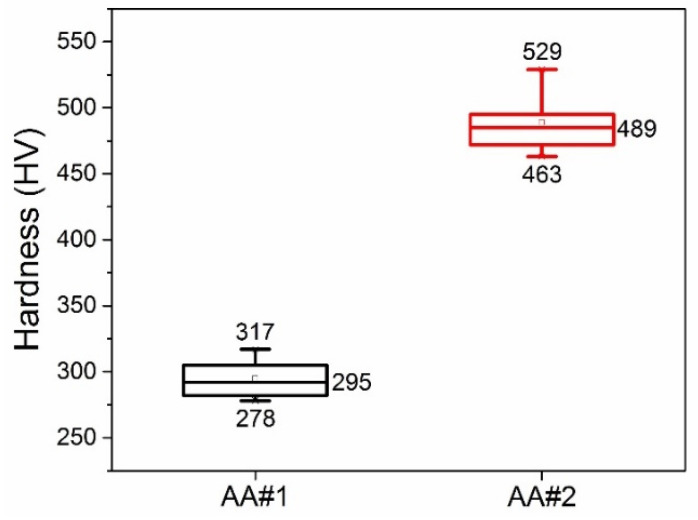
Microhardness of AAO measured by the Vickers method for AA#1 and AA#2 aluminum alloys (applied load: 100 g).

The result of measuring the micro hardness by the Vickers method confirmed differences in the mechanical properties of two types of AAO obtained on different aluminum alloys. To study the microhardness, samples were taken of each aluminum alloy and anodized with stabilization at a voltage of 120 V and a temperature of 22 °C. For each sample, the microhardness was measured at five points. Figure 3 shows integral statistical data of the micro hardness values of AAO layers based on AA#1 and AA#2 alloys. It can be seen that for the AA#1 alloy, the median value is about 295 HV, and for AA#2, it is about 489 HV. The hardness of AAO based on the AA#2 alloy is about 50% higher than the hardness of AAO based on the AA#1 alloy. The results of measuring the micro hardness of AAO confirmed our conclusion that the determining factor in the mechanical strength of the obtained AAO is the alloy used, and not the anodizing mode.

**Figure 4:**
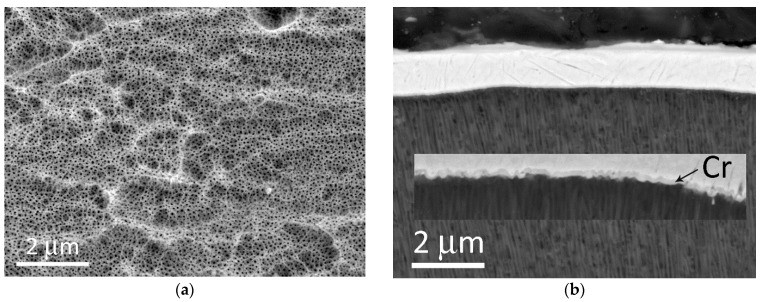
(**a**) Plan view of unpolished AAO without metallization. (**b**) Cross-section view of unpolished AA#1 with Cr and Cu layers deposited by magnetron sputtering.

SEM studies of the AAO microstructure were carried out to explain the different mechanical properties of AAO based on two aluminum alloys. Figure 4a is a top view of an unpolished AAO. A surface is not planar and represented by “hills” and “pits”, formed by initial morphology before the anodizing process and by etching inclusions during the anodizing. A micrograph of a cross-section of anodized AA#1 with a deposited adhesion layer of chromium of 100–120 nm and subsequent deposition of copper with a thickness of 1 μm is shown in Figure 4b. The inset shows a high-magnification image, where (1) the penetration of chromium into the pores and (2) the formation of a zigzag profile of the chromium sublayer are observed. In the case of the unpolished AAO surface, upon further galvanic deposition of copper to a thickness of 300 μm, the median value of pull-off adhesion was 5–6 N/mm^2^ for both aluminum alloys.

It was shown in [18] that the adhesion force of a nanofilm to a rough substrate substantially depends on the geometric parameters of the substrate, such as the wavelength and amplitude of the roughness, as well as the thickness of the adhesion layer. Therefore, to improve the adhesion of the 300 μm copper layer, the surface of the workpiece with the AAO layer was planarized in a grinding machine. The mechanical properties of the AAO turned out to be critical at the surface planarization process and subsequent adhesion tests.

**Figure 5:**
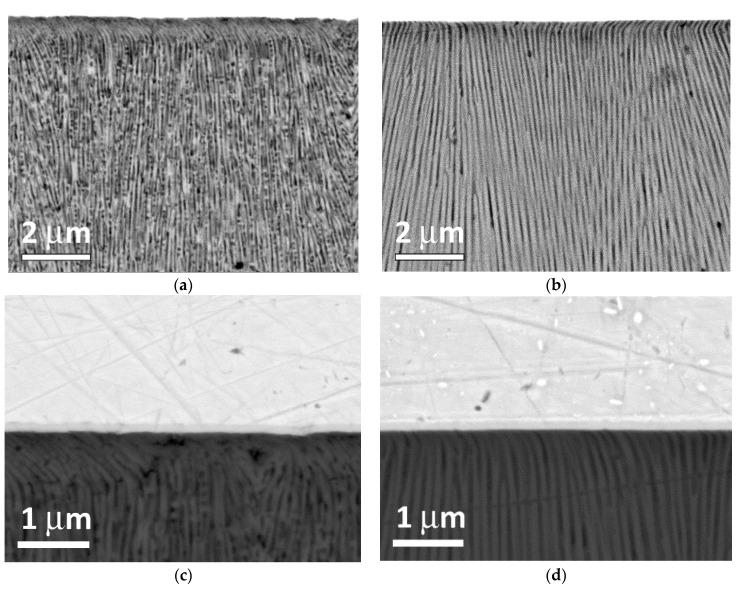
(**a**,**c**) Cross-section view of polished AA#1. (**b,d**) Cross-section view of polished AA#2.

Figure 5a,c show cross-sectional images of a polished AAO sample based on the AA#1 alloy. The porous structure is represented by extended vertical pores with a diameter and wall thickness of about 100 nm. It is worth noting that there are spherical holes with a diameter of about 70–80 nm in the walls observed in alumina structure of AA#1. The formation of these holes is discussed below. It should be noted that in the case of AAO based on AA#1, the upper layer of the porous structure is damaged during abrasive polishing to a depth of 1 μm. Moreover, at high magnifications, the rupture of the AAO walls is clearly visible, which is also a factor that reduces the results of measuring the adhesive/cohesive strength of anodized alumina. In the case of AAO based on the AA#2 alloy (Figure 5b,d), only extended pores are observed, with a pore diameter and wall thickness of the AAO similar to the AA#1 alloy. A detailed examination of the AAO/Cr interface shows that the thickness of the deformed near-surface layer is only 100–300 nm. A detailed study of this interface on various AAO samples on AA#2 did not show any breaks in the alumina walls; only bending was identified. The shape and behavior of the near-surface alumina layer on AA#2 alloy seems to be less fragile than AAO on AA#1. We suggest that spherical holes in the walls of the alumina based on AA#1 are the main reason for less resistance to machining and mechanical tests such as planarization, hardness and adhesion measurements.

The analysis of the structure of the oxide was also carried out on two additional samples, AA#3 with a copper content of 0.008% and AA#4 with a copper content of less than 0.002%, a detailed chemical analysis of which is given in Table S1 in the Supplementary Materials. The study of the structure in SEM showed that the defect-free anodic oxide porous structure was preserved in the near-surface region and near the interface with bulk aluminum for in both samples (see Figure S1a–d). In the case of sample AA#2, the defect free porous structure was observed in the near-surface layer of the order of 30 μm. In deeper layers, the structure of the anodic oxide became similar to sample AA#1 (see Figure S1e–h).

Let us consider the probable reason for the difference in the properties of AAO formed on different aluminum alloys with Al content higher than 99.3 wt %. It is known that in the process of anodizing an aluminum alloy, only aluminum is anodized with the formation of AAO. In the initial period of anodic oxidation, an ideal process is observed, in which only chemically pure aluminum is involved, and the alloying components, in essence, are not capable of anodizing [19]. Due to this phenomenon, at the same time, the aluminum alloy is enriched with alloying elements in a layer 1–5 nm thick, located directly under the anode film [20,21]. Furthermore, when the concentration of alloying components reaches a critical value, they begin to oxidize or dissolve (depending on the element), thereby leading to the formation of a loose and porous structure of the oxide walls. Comparing the chemical composition of the initial aluminum alloys, we find a significant discrepancy only in the copper content. In AA#1, its content is about 0.15% by weight, and for AA#2, it is 0.06%. The presence of copper as the main alloying element and associated constituent particles and conglomerates formed during heat aging treatment increases the mechanical strength of the alloy, but unfortunately stimulates galvanic corrosion due to different electrochemical potentials between the matrix and dispersed intermetallids [19,21]. Thus, due to the high content of copper in the AA#1 alloy, the critical value of its concentration occurs almost immediately after the start of anodizing. In the case of AA#2, the critical concentration of copper under the anode film is reached only at a depth of 20–30 µm, and the structure of the AAO of both alloys becomes similar. In alloys with an ultra-low copper content, the formation of spherical holes is not observed throughout the thickness of the anodic aluminum oxide.

## 4. Conclusions

The work investigated the mechanical properties of AAO obtained on two similar aluminum alloys with 99.3% content of Al, but with different impurity ratios. In the case of AAO on alloy AA#2, the pull-off adhesion of 300 μm copper metallization was 27 N/mm^2^ and the peel test adhesion of 100 μm copper was 5.5 N/mm; for AA#1, they were 12 N/mm^2^ and 2.5 N/mm, respectively. The study of micro hardness showed that in the case of AAO based on AA#2, the hardness was 498 HV, 50% higher than the hardness of the alumina on AA#1. SEM studies of the AAO structure revealed the presence of isolated single pores, leading to the fragility of AAO. The presence of these pores, appears to be due to the increased concentration of copper, being about 0.15 wt % in the AA#1 alloy compared with 0.06 wt % in AA#2.

The obtained values of the adhesion strength of cooper metallization to AAO from the generally available aluminum alloys of A1100 series with reduced copper content meet the world requirements for DBC structures. The introduction of composite materials based on anodized aluminum into the packaging of power modules can be technologically and economically justified.

## Figures and Tables

**Table 1:** The weight elemental composition of investigated aluminum alloys.

Alloy	Al	Si	Fe	Cu	Mn	Mg	Cr	Zn	Ti	Ni
AA#1	99.3	0.107	0.380	0.147	0.004	0.006	0.002	0.008	0.01	0.003
AA#2	99.5	0.095	0.252	0.057	0.006	0.015	0.002	0.008	0.020	0.005

## Data Availability

Initial experimental data can be obtained upon request from the corresponding author, O.S. Medvedev.

## Supplementary Materials

The following are available online at https://www.mdpi.com/article/10.3390/ma14227028/s1, Table S1: The weight elemental composition of investigated aluminum alloys, Figure S1: Scanning electron images of cross section AA#4 (a, b), AA#3 (c, d), AA#2 (e, f) and AA#1 (g, h). SEM images (a, c, e and g) obtained near the surface of anodic aluminum oxide, images (b, d, f and h)—near the interface of anodic aluminum oxide and bulk aluminum.
