# Supplementary material for: Influence of Alloying Elements on the Mechanical Properties of Anodized Aluminum and on the Adhesion of Copper Metallization
Source: Materials (Basel). 2021 Nov 19;14(22):7028. doi: 10.3390/ma14227028 (PMC8624961; doi:10.3390/ma14227028)
Supplement: Supplementary file 1 [file materials-14-07028-s001.zip › materials-1445985-supplementary.pdf]

Supplementary material

Influence of Alloying Elements on the Mechanical Properties of Anodized Aluminum and on the Adhesion of Copper Metallization

Oleg S. Medvedev [1,*], Ekaterina E. Alyasova [1], Rona E. Besprozvannaya [1], Asadula A. Gadzhiev [1], Veronika V. Krivova [1], Andrey S. Kondratev [1], Artem E. Kim [2], Pavel A. Novikov [2] and Anatoliy A. Popovich [2]

[1] LLC "Rusoxide", 143026 Skolkovo, Moscow, Russia; aee@rusoxide.ru (E.E.A.); bre@rusoxide.ru (R.E.B.); gaa@rusoxide.ru (A.A.G.); kvv@rusoxide.ru (V.V.K.); kas@rusoxide.ru (A.S.K.)

[2] Peter the Great Saint-Petersburg Polytechnic University, 195251 Saint Petersburg, Russia; artem_7.kim@mail.ru (A.E.K.), novikov_pa@spbstu.ru (P.A.N.) director@immet.spbstu.ru (A.A.P.)

* Correspondence: medvedev.os1990@gmail.com

Table S1. The weight elemental composition of investigated aluminum alloys.

Alloy	Al	Si	Fe	Cu	Mn	Mg	Cr	Zn	Ti	Ni
AA#1	99.3	0.107	0.380	0.147	0.004	0.006	0.002	0.008	0.01	0.003
AA#2	99.5	0.095	0.252	0.057	0.006	0.015	0.002	0.008	0.020	0.005
AA#3	99.5	0.041	0.279	0.008	0.005	0.005	0.003	0.006	0.134	0.008
AA#4	99.99	<0.003	<0.003	<0.002	<0.002	<0.001	<0.002	<0.002	<0.002	<0.002

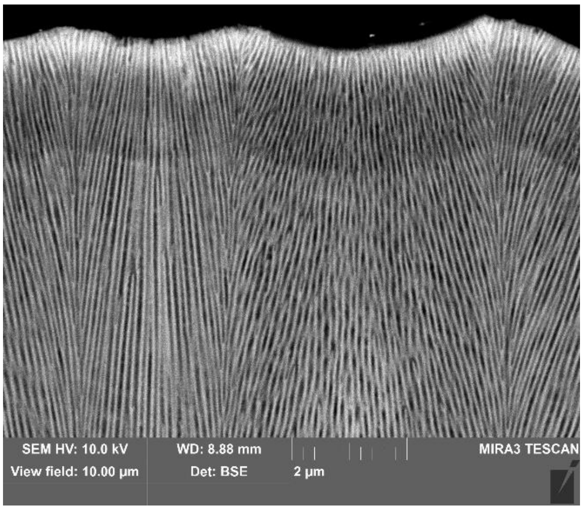
SEM HV: 10.0 kV
WD: 8.88 mm
MIRA3 TESCAN
View field: 10.00 μm
Det: BSE
2 μm

(a)

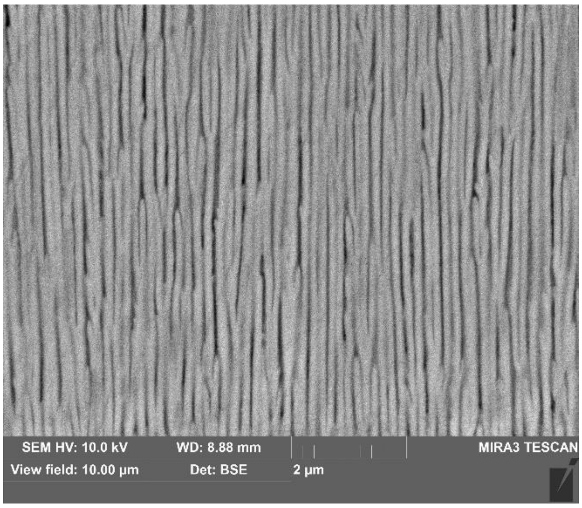
SEM HV: 10.0 kV
WD: 8.88 mm
MIRA3 TESCAN
View field: 10.00 μm
Det: BSE
2 μm

(b)

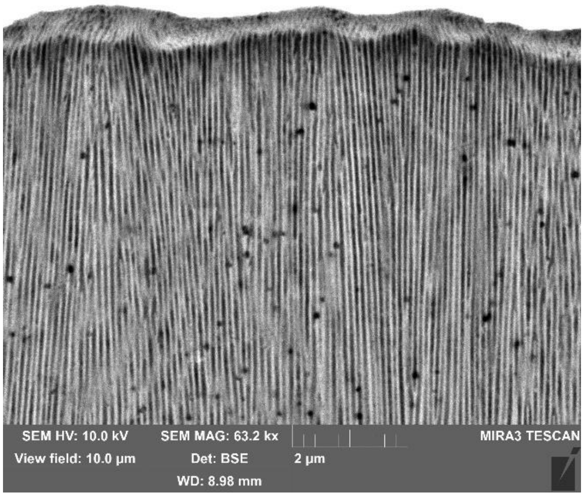
SEM HV: 10.0 kV
SEM MAG: 63.2 kx
MIRA3 TESCAN
View field: 10.0 μm
Det: BSE
2 μm
WD: 8.98 mm

(c)

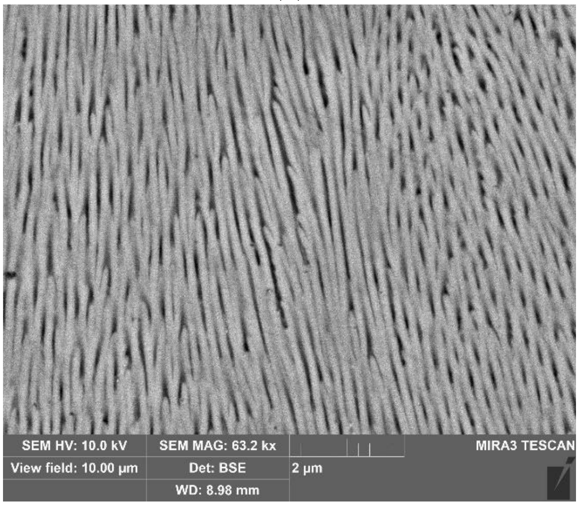
SEM HV: 10.0 kV
SEM MAG: 63.2 kx
MIRA3 TESCAN
View field: 10.00 μm
Det: BSE
2 μm
WD: 8.98 mm

(d)

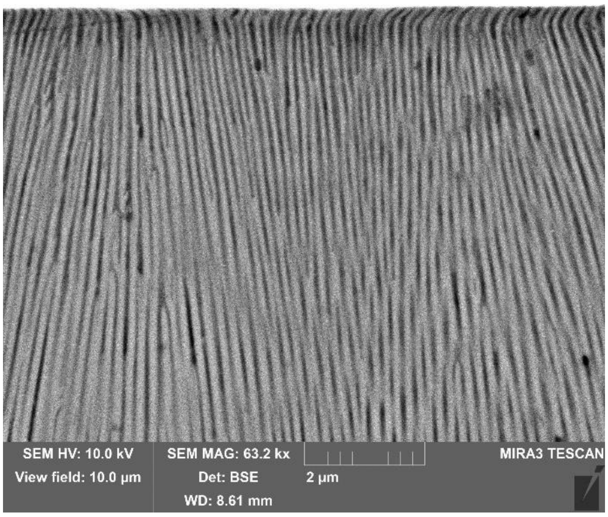
SEM HV: 10.0 kV
SEM MAG: 63.2 kx
MIRA3 TESCAN
View field: 10.0 μm
Det: BSE
2 μm
WD: 8.61 mm

(e)

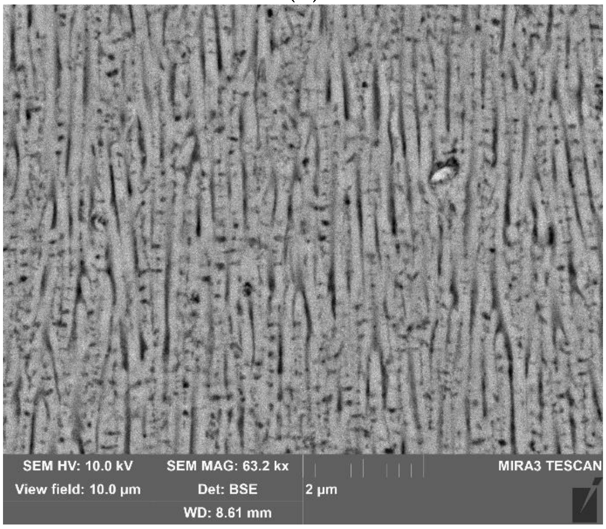
SEM HV: 10.0 kV
SEM MAG: 63.2 kx
MIRA3 TESCAN
View field: 10.0 μm
Det: BSE
2 μm
WD: 8.61 mm

(f)

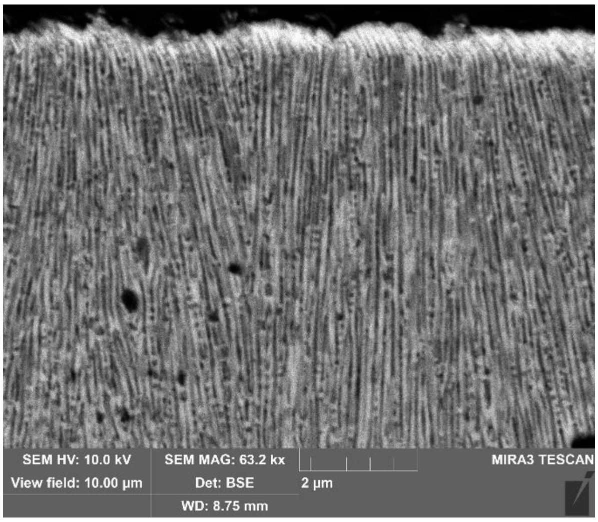

(g)

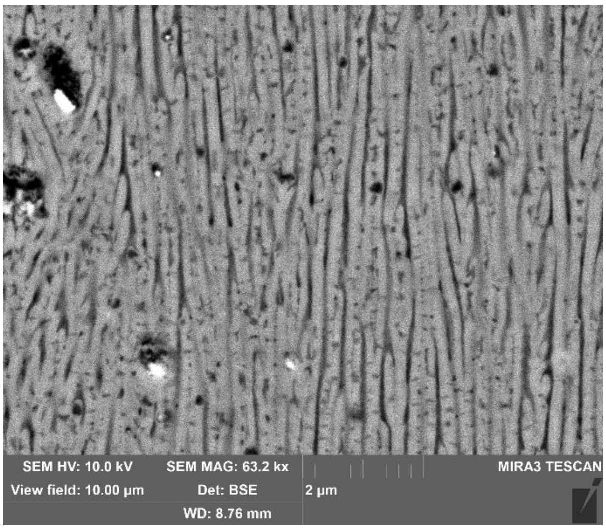

(h)

Figure S1. Scanning electron images of cross section AA#4 (**a**, **b**), AA#3 (**c**, **d**), AA#2 (**e**, **f**) and AA#1 (**g**, **h**). SEM images (**a**, **c**, **e** and **g**) obtained near the surface of anodic aluminum oxide, images (**b**, **d**, **f** and **h**) – near the interface of anodic aluminum oxide and bulk aluminum.
